# Conjunctival Inflammation in Thrombospondin-1 Deficient Mouse Model of Sjögren’s Syndrome

**DOI:** 10.1371/journal.pone.0075937

**Published:** 2013-09-23

**Authors:** Laura Contreras-Ruiz, Birgit Regenfuss, Fayaz Ahmad Mir, James Kearns, Sharmila Masli

**Affiliations:** Schepens Eye Research Institute and Massachusetts Eye and Ear, Department of Ophthalmology, Harvard Medical School, Boston, Massachusetts, United States of America; Center for Cancer Research, National Cancer Institute, United States of America

## Abstract

Lacrimal gland inflammation during autoimmune Sjögren’s syndrome (SS) leads to ocular surface inflammation – Keratoconjunctivitis sicca (KCS). This condition afflicts both the cornea and conjunctiva that form the ocular surface. Thrombospondin-1 (TSP-1) deficiency in mice results in lacrimal gland and corneal inflammation that resembles the human disease. In this study we report conjunctival pathology in this mouse model of SS. We found that TSP-1 null mice develop inflammation in the conjunctiva and associated loss of goblet cell function similar to that seen in patients with SS. Increased expression of Th1 (IFN-γ, TNF-α) and Th17 (IL-6, IL-17A) inflammatory cytokines and related transcription factors (Tbet and RORγt) were detected in TSP-1 null conjunctiva as well as their draining lymph nodes (LNs). The conjunctival inflammation was also accompanied by an increase in local lymphatic vessels. Interestingly, migration of antigen-bearing dendritic cells (DCs) from the ocular surface to the LNs was dependent on the TSP-1 available in the tissue. These results not only reveal potential immunopathogenic mechanisms underlying KCS in SS but also highlight the therapeutic potential of TSP-1.

## Introduction

Sjögren’s syndrome (SS) is the second most common autoimmune rheumatic disease and afflicts an estimated 2-4 million individuals in the United States [1]. It is an autoimmune exocrinopathy involving chronic inflammation and progressive functional loss of exocrine glands – salivary and lacrimal glands. The secretory deficit of these glands consequently affects the target organ protected by their secretions resulting in clinical manifestations of xerostomia (dry mouth) and xerophthalmia (dry eye). The latter condition is also referred to as Keratoconjunctivitis sicca (KCS) indicating the dryness and inflammation of the cornea and conjunctiva of the eye that form the ocular surface. Patients with Sjögren’s syndrome are reported to develop inflammatory infiltrates in the salivary and lacrimal glands [2], a pathology that is well reproduced in mice known to develop the disease spontaneously [3-5]. Pathologic changes are also detectable in the conjunctiva of patients with Sjögren’s syndrome [6-8], however these are not yet reported in any of the mice with this disease. An animal model that recapitulates most pathologic features of the disease can serve as a useful tool not only to reveal underlying mechanisms but also to develop comprehensive therapeutic approaches that target multiple pathologic aspects of the disease.

We have previously reported spontaneous development of autoimmune SS-associated ocular phenotype in thrombospondin-1 (TSP-1) deficient mice that closely resembles human disease [9]. As seen in Sjögren’s patients these mice develop KCS progressively with age, which coincides with a secretory deficit of lacrimal glands and altered tear composition resulting in a gradual loss of corneal barrier function. Moreover anti-SSA and anti-SSB autoantibodies that develop in Sjögren’s patients are also detectable in TSP-1 deficient mice. Thus, to our knowledge, these mice represent the only mouse model in which chronic development of ocular surface inflammation associated with autoimmune SS is reported.

Thrombospondin-1 is a platelet derived glycoprotein that is also expressed by other cell types including epithelial cells of the ocular tissues [10,11]. Besides its ability to activate latent TGF-β [12], TSP-1 is reported to be involved in regulation of vascular and lymphatic angiogenesis [13]. It has been described to have an immunoregulatory function with anti-inflammatory effects on antigen presenting cells and immune effector T cells [14-17]. We have reported previously that expression of TSP-1 is critical for the ocular immune privilege that protects ocular tissues from sight threatening inflammatory damage [18]. The immunomodulation induced by ocular antigen presenting cells is dependent on their expression of TSP-1 [19]. Consistent with these reports a significant decline in the splenic population of regulatory T cells is observed in TSP-1 deficient mice [9] indicative of failure of peripheral immune tolerance, which presumably contributes to the autoimmunity in these mice. Similar to Sjögren’s syndrome in human patients [20], the autoimmune response in TSP-1 deficient mice targets the lacrimal gland resulting in infiltration with Th1 and Th17 inflammatory subsets associated with autoimmune diseases. Infiltration of the conjunctiva by T cells was reported in human subjects diagnosed with KCS [21]. However, unlike lacrimal gland infiltrates, any subset of T cells in conjunctival infiltrates still remain to be characterized. None of the currently available SS mouse models have reported conjunctival pathology.

In this study we now report that the inflammation in the conjunctiva is involved in the KCS present in the TSP-1 deficient mouse model of SS, which potentially disrupts the secretory function of goblet cells further compromising the protective function of tears and contributing to ocular surface damage. We evaluated the temporal association of conjunctival inflammation with the inflammatory effectors in the draining lymph nodes of TSP-1 null mice. Our results suggest that enhanced migration of ocular surface antigen presenting cells (APCs) to draining lymph nodes, in TSP-1null mice, potentially contributes to the induction of immune response against antigens derived from the ocular surface. Moreover, TSP-1 was found to regulate expression of a lymph node homing receptor, CCR7, on dendritic cells (DC) and successfully blocked DC migration to draining lymph nodes when injected subconjuctivally in TSP-1 deficient mice. Thus our results demonstrate that TSP-1null mice mimic conjunctival manifestations of autoimmune Sjögren’s syndrome and reveal a potential immunopathogenic mechanism to be targeted by new therapeutic approaches to help prevent progression of the disease.

## Materials and Methods

### Ethics Statement

All animal experiments were conducted in accordance with institutional guidelines. The protocol (S-249-0912) was approved by the Institutional Animal Care and Use Committee (IACUC) at Schepens Eye Research Institute. All efforts were made to minimize suffering including the use of ketamine:xylazine anesthesia administered prior to performing s.c. injections.

### Mice

C57BL/6 (*H-2b*) mice, 4 to 12 weeks old, were purchased from Charles River Laboratories (Wilmington, MA). TSP-1 null mice (C57BL/6 background), originally received from Dr. J. Lawler (BIDMC, Harvard Medical School, Boston, MA) were bred in-house in a pathogen-free facility at Schepens Eye Research Institute, Boston, MA.

### Histology

Lacrimal glands or whole eyes and lids were harvested and fixed immediately in 4% paraformaldehyde and embedded in methacrylate. Sections (6 to 8 µm) were cut and stained with H&E. For the sections of the conjunctiva we used sagittal sections from the middle of the eye as representative of the whole conjunctiva to control for the variations in goblet cell density over the surface of the eye. Both the bulbar and palpebral conjunctiva were examined on three slides with five serial sections each.

### Immunohistochemistry

TSP-1 null and WT C57BL/6 mice were euthanized at 8 or 12 weeks of age. The conjunctivas were harvested, rinsed in PBS, fixed in acetone and washed in PBS. Then the tissue was incubated in EDTA and rinsed in PBS again. After blocking with BSA conjunctival flat mounts were incubated in anti-LYVE-1 antibody (AngioBio, Del Mar, CA) overnight. The next day the tissue was washed, blocked and incubated with a Cy3-conjugated secondary antibody (Jackson ImmunoResearch, West Grove, PA). After rinsing in PBS the tissue was incubated in Alexa488-conjugated CD11b antibody (BD Biosciences, San Jose, CA). Then the flat mounts were washed in PBS, transferred to glass slides, coverslipped with mounting medium and stored at 4°C prior to analysis with a fluorescence microscope. Three pictures were randomly taken of each flat mount with a fluorescence microscope, the LYVE-1 and CD11b positive cells were counted manually and the percentage of the Lyve-1^+^ / CD11b^+^ cells to all CD11b^+^ cells was calculated.

## ELISA

Pilocarpine-induced tears in some experiments were measured for their levels of MUC5AC content using an ELISA kit (TSZ ELISA, Waltham, MA). The assay was performed according to the manufacturer’s instructions.

### Real-time PCR

Total RNA was isolated from the conjunctiva or cervical lymph nodes harvested from WT or TSP-1 null mice using RNA STAT-60 (Tel-Test, Inc., Friendswood, TX) according to the manufacturer’s instructions. cDNA was synthesized by reverse transcribing RNA using oligo dT and M-MLV RT (Promega, Madison, WI). SYBR green real-time PCR assay was used to determine relative quantitative expression of selected genes. Sequences of the primers used for these genes are as follows: *glyceraldehyde-3-phosphate dehydrogenase*, F-5’-CGAGAATGGGAAGCTTGTCA-3’, R-5’-AGACACCAGTAGACTCCACGACAT-3’. *Interferon-gamma*, F-5’-TCAGCAACAACATAAGCGTCAT-3’, R-5’-GACCTCAAACTTGGCAATACTCAT-3’, *Interleukin-6*, F-5’-AGTCAATTCCAGAAACCGCTATGA-3’, R-5’-TAGGGAAGGCCGTGGTTGT-3’, *Interleukin-17A*, F-5’-AGTGAAGGCAGCAGCGATCAT-3’, R-5’-CGCCAAGGGAGTTAAAG-3’, *RORγt*, F-5’-TCAAGTTTGGCCGAATGTC-3’, R-5’-CATCTGAGAGCCCTAAAGTGTATG-3’, *Tbet*, F-5’-CGCATCTGTTGATACGAGTGT-3’, R-5’-GCTGGGAACAGGATACTG-3’, *FoxP3*, F-5’-GGAGAGGCAGAGGACACTCAAT-3’, R-5’-GTGGTTTCTGAAGTAGGCGAACAT-3’, *MUC5AC*, F-5’- CCAACTCCAGCCATATCGT -3’, R-5’- CAGGGTAACTGCCATCTAGC -3’. Amplification reactions were set up using KAPA SYBR FAST mastermix (KAPA Biosystems, Woburn, MA) in triplicates with the thermal profile: 1 cycle of 95°C for 5 min; 40 cycles of [95°C for 10 seconds, 52°C to 55°C for 10 seconds, 72°C for 10 seconds] on an Eppendorf Realplex2 (Eppendorf AG, Hamburg, Germany). Fluorescence signal generated at each cycle was analyzed using system software. The threshold cycle values were used to determine relative quantitation of gene expression with glyceraldehyde-3-phosphate dehydrogenase as a reference gene.

### Draining of ocular surface antigen to cervical lymph nodes

Two groups of TSP-1 null mice (n=3 each) received either a subconjunctival injection of 10 µl TSP-1 (Haematologic Technologies, Essex Junction, VT) in PBS (50 ng/10 µl, per eye) or PBS alone (10 µl per eye). A control group of WT mice received no injections. A day after subconjunctival injections all mice received eye drops containing Alexa647-conjugated Ovalbumin (Life Technologies, Carlsbad, CA) in PBS (250 µg/5 µl per mouse, 2.5 µl per eye). After 3 h all the mice were sacrificed and their cervical lymph nodes (CLNs) were collected. The CLNs in each group were pooled, filtered through a 70µm nylon mesh, and the resulting single-cell suspensions were washed and resuspended in cold PBS containing 0.1% bovine serum albumin. Cells from CLNs were stained with eFluor 780-conjugated Fixable Viability Dye (eBioscience, San Diego, CA) and fluorescence labeled anti-CD11c (BD Biosciences, San Jose, CA).

### Flow cytometry

Dendritic cells were generated by culturing bone marrow cells (10 x 10^6^) with granulocyte-macrophage colony-stimulating factor (GM-CSF) (20 ng/ml, Biolegend, San Diego, CA) in a petri dish for 7 days. The medium in these cultures was replenished every 2 days. Bone marrow derived dendritic cells (BMDCs) were treated with TSP-1 (1 µg/ml, Hematologic Technologies, Inc., VT) for 24 h before staining with fluorescence labeled anti-CCR7 antibody (eBioscience, San Diego, CA). Fluorescence-labeled cells were analyzed using a BD LSR II flow cytometer (BD Biosciences, San Jose, CA). Further analysis of the data was performed using FlowJo software.

### Statistical Analysis

Student’s unpaired t-test was used to determine significant differences between mean values of experimental and control groups. Error bars in figures represent + SEM and p < 0.05 was considered statistically significant.

## Results

### Inflammatory damage is detectable in TSP-1 null conjunctiva

Deficiency of TSP-1 was previously reported to result in an appearance of inflammatory infiltrate in the lacrimal gland [9]. Such infiltration was detected at 24 weeks of age while no significant inflammatory cells were detectable in the lacrimal gland histology of younger animals. To determine if ocular inflammation in TSP-1 deficient mice is associated with any inflammatory damage in the conjunctiva we examined conjunctiva histology. Tissues sections stained with H&E were compared at different ages.

**Figure 1:**
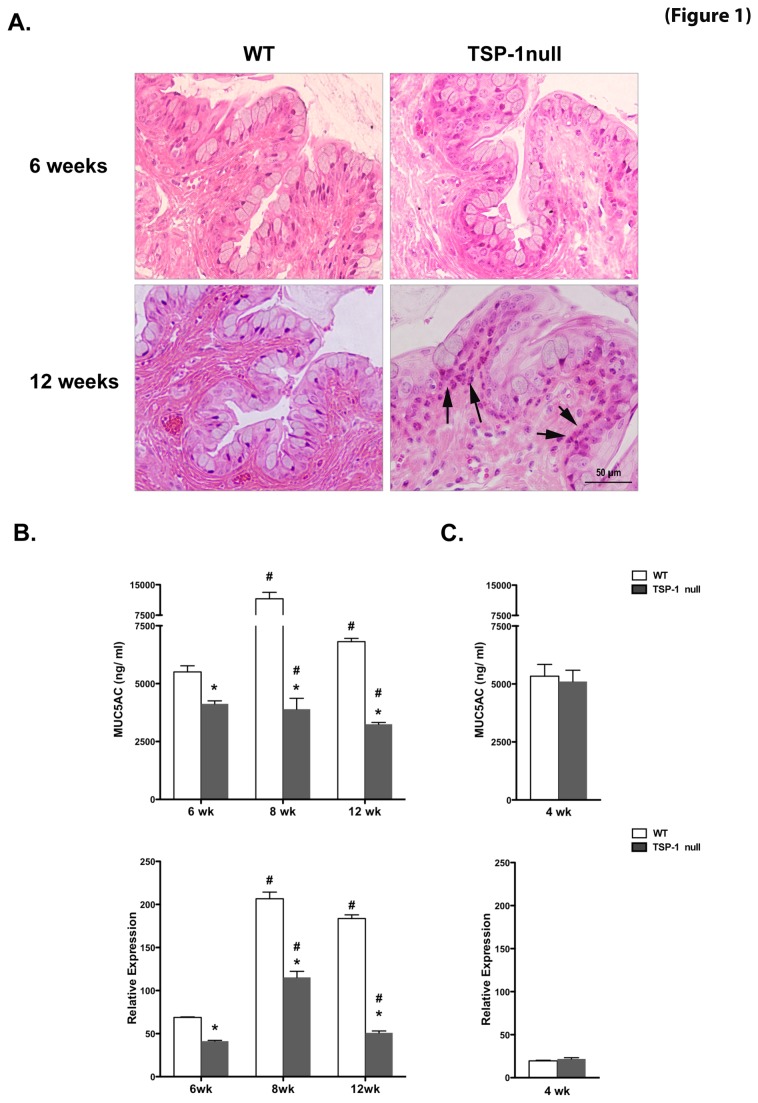
Inflammatory damage and diminished mucin levels are detectable in TSP-1 null conjunctiva. (**A**) Histology of conjunctiva from WT and TSP-1 null mice at 6 and 12 weeks. Representative hematoxylin-eosin stained sections at 6 weeks show normal epithelial morphology and a lack of abnormal inflammatory cells. At 12 weeks, TSP-1 null conjunctiva displayed prominent inflammatory infiltrates (arrows) in the epithelial layer adjacent to goblet cells. (**B**-**C**) Pilocarpine-induced tears and conjunctival tissues collected from WT or TSP-1 null mice (n=5 each) were analyzed for levels of MUC5AC using ELISA and real time PCR, respectively. At 6, 8, and 12 weeks of age, goblet cell-derived tear mucin content and conjunctival message were significantly diminished in TSP-1 null mice compared to age-matched controls, while no significant differences were detected at 4 weeks of age. Results are presented as ng/ml for ELISA assay and as relative expression to that of housekeeping gene GAPDH for real time PCR (*p < 0.05 compared to WT control; #p < 0.05 compared to 4 weeks).

As shown in Figure 1A, at 6 weeks of age TSP-1 null conjunctiva was comparable to that in WT control and showed normal cell layers and morphology with abundant conjunctival epithelial goblet cells and a complete lack of abnormal inflammatory cells. However, at 12 weeks, TSP-1 null conjunctiva displayed a marked thickening of the outer epithelial layers, a relative decrease in goblet cell numbers and a prominent infiltration of inflammatory cells, while no such alterations were detectable in WT conjunctiva.

These results suggest that ocular inflammation in TSP-1 null mice involves inflammatory damage of the conjunctiva and that this damage is histologically detectable at 12 weeks of age. Thus inflammatory changes in the conjunctiva in TSP-1 null mice appear to precede those reported in their lacrimal glands.

### Tear mucin levels are diminished in TSP-1 null mice

Mucins are an important and protective component of tears. While both the membrane bound and soluble forms of mucins are produced by epithelial cells in the conjunctiva, a gel-forming soluble mucin MUC5AC is secreted by goblet cells in the conjunctiva. To determine whether the histopathological finding in TSP-1 null conjunctiva was associated with secretory impairment of goblet cells, we assessed changes in levels of MUC5AC in pilocarpine-induced tears and its message in conjunctiva, with age.

As seen in Figure 1B, a significant decrease in the MUC5AC content of tears was detected at 12, 8, and 6-week-old TSP-1 deficient mice compared to their age-matched WT controls. However, no significant differences were detected at 4 weeks of age. These changes matched those detected in the MUC5AC message level (Figure 1C). It is to be noted that tear MUC5AC levels progressively decreased with age in TSP-1 null mice.

Therefore these results suggest that the secretory function of goblet cells in the conjunctiva, despite appearing normal at 4 weeks, becomes progressively deficient with age in the absence of TSP-1. The functional loss of goblet cells in TSP-1 null mice precedes the histopathology detected.

### Inflammatory cytokines are detectable in TSP-1 null conjunctiva in the absence of apparent inflammatory infiltrates

Although the presence of inflammatory infiltrate in the target tissue is considered a primary immunopathogenic indicator, the microscopic exam of tissue histology limits detection of early changes that lead to the infiltration. Assessing changes in the expression of inflammatory cytokines by real-time PCR allows detection of changes prior to the appearance of infiltrates. Thus in TSP-1 deficient mice increased levels of inflammatory cytokines were detectable in lacrimal glands long before the inflammatory infiltrates were detectable [9]. Considering that the secretory changes in conjunctival goblet cells begin prior to the detection of infiltrates, we performed real-time PCR analysis on the RNA isolated from WT or TSP-1 null conjunctiva to study expression levels of dry eye-associated inflammatory cytokines.

**Figure 2:**
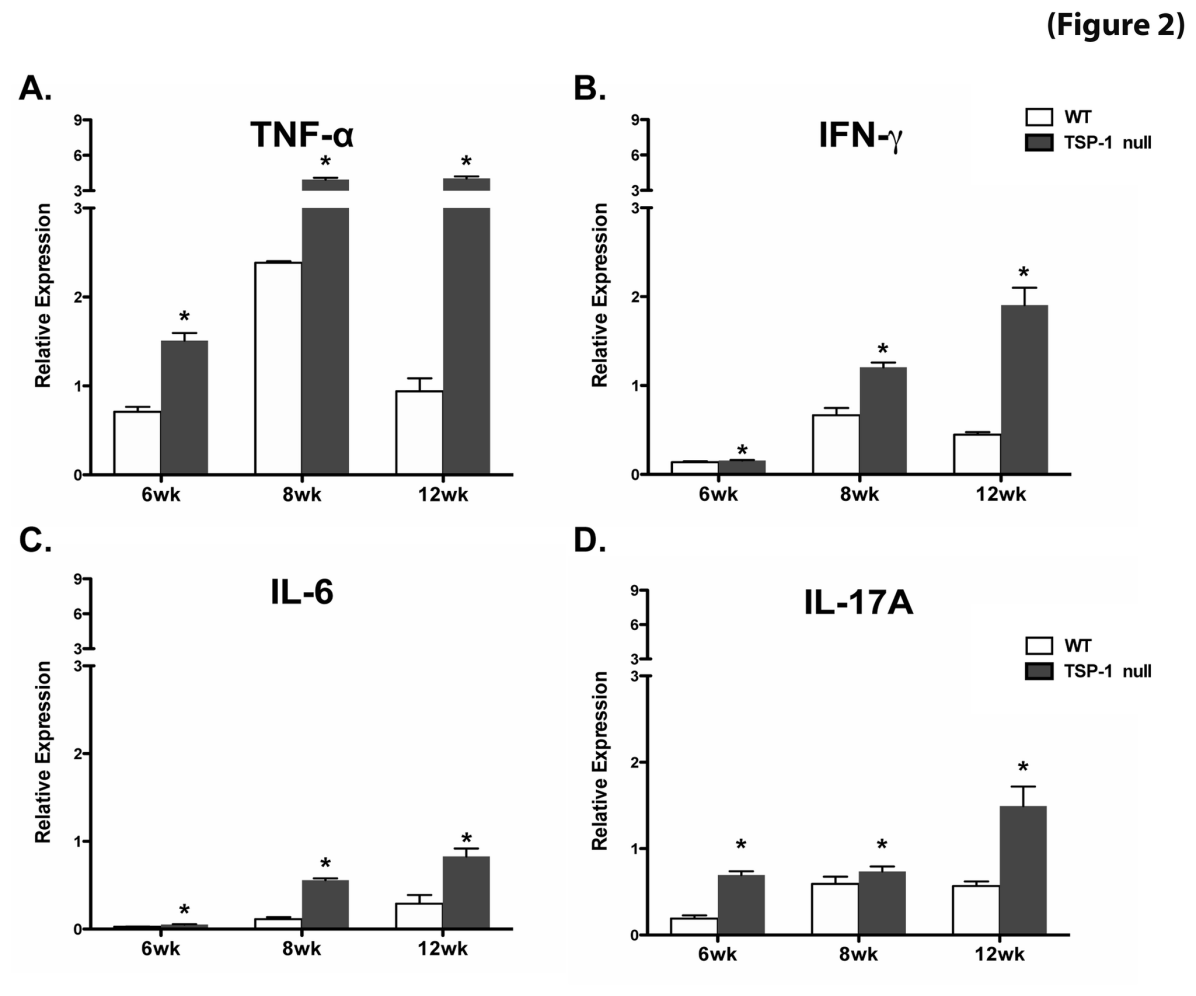
Inflammatory cytokines are detectable in TSP-1 null conjunctiva in the absence of apparent inflammatory infiltrates. Conjunctiva tissue from WT and TSP-1 null mice was harvested at 6, 8, and 12 weeks (n=3 each) to isolate RNA, which was analyzed using a SYBR Green real-time PCR assay to determine levels of INF-γ (**A**), TNF-α (**B**), IL-6 (**C**) and IL-17A (**D**). Significantly increased expression of Th1 (TNF-α, IFN-γ) and Th17 (IL-6 and IL-17A) cytokines was detected in TSP-1 null conjunctiva at all tested ages, compared to their age-matched WT control tissue. The expression of all studied cytokines progressively increased with age. Results are presented as relative expression of a cytokine gene to that of housekeeping gene GAPDH (*p < 0.05 as compared to WT controls)..

Significant over expression of Th1 (TNF-α, IFN-γ) and Th17 (IL-6 and IL-17A) cytokines was detected in TSP-1 null conjunctiva harvested from 6, 8, and 12 week old mice compared to the age-matched WT control tissue. The over expression of all studied cytokines progressively increased with the age of the mice (Figure 2).

Our findings suggest that TSP-1 deficiency in mice results in inflammatory damage of the conjunctiva as reported in patients with KCS associated with SS [22,23]. Moreover, our results also indicate that the inflammatory changes begin as early as 6 weeks, and are gradually aggravated with age. These changes appear to coincide with the loss of MUC5AC secretion by conjunctival goblet cells.

### TSP-1 deficiency results in increased lymphatic vessels in the conjunctiva

Lymphatic vessels play an important role in any disease immunopathogenesis, providing a potential route by which tissue antigen presenting cells (APCs) can introduce tissue–derived antigens into the draining lymph nodes. In an otherwise avascular tissue such as cornea the association of lymphangiogenesis with inflammation has been reported and TSP-1 is known to inhibit such inflammatory lymphangiogenesis [13]. In addition, it has been previously described that during corneal inflammation an increase in lymphatic vessels corresponds to a decline in the conjunctival population of LYVE-1 expressing monocytic cells suggesting such cells to be a reservoir for lymphangiogenic cells [24]. Thus the number of LYVE-1^+^ monocytic cells can be an indirect measure of lymphatic vessel density. To examine whether TSP-1 deficiency is associated with changes in conjunctival lymphangiogenesis, we first evaluated lymphatic vessels by histology and then by enumerating LYVE-1 expressing monocytic cells by immunoflourescence studies.

**Figure 3:**
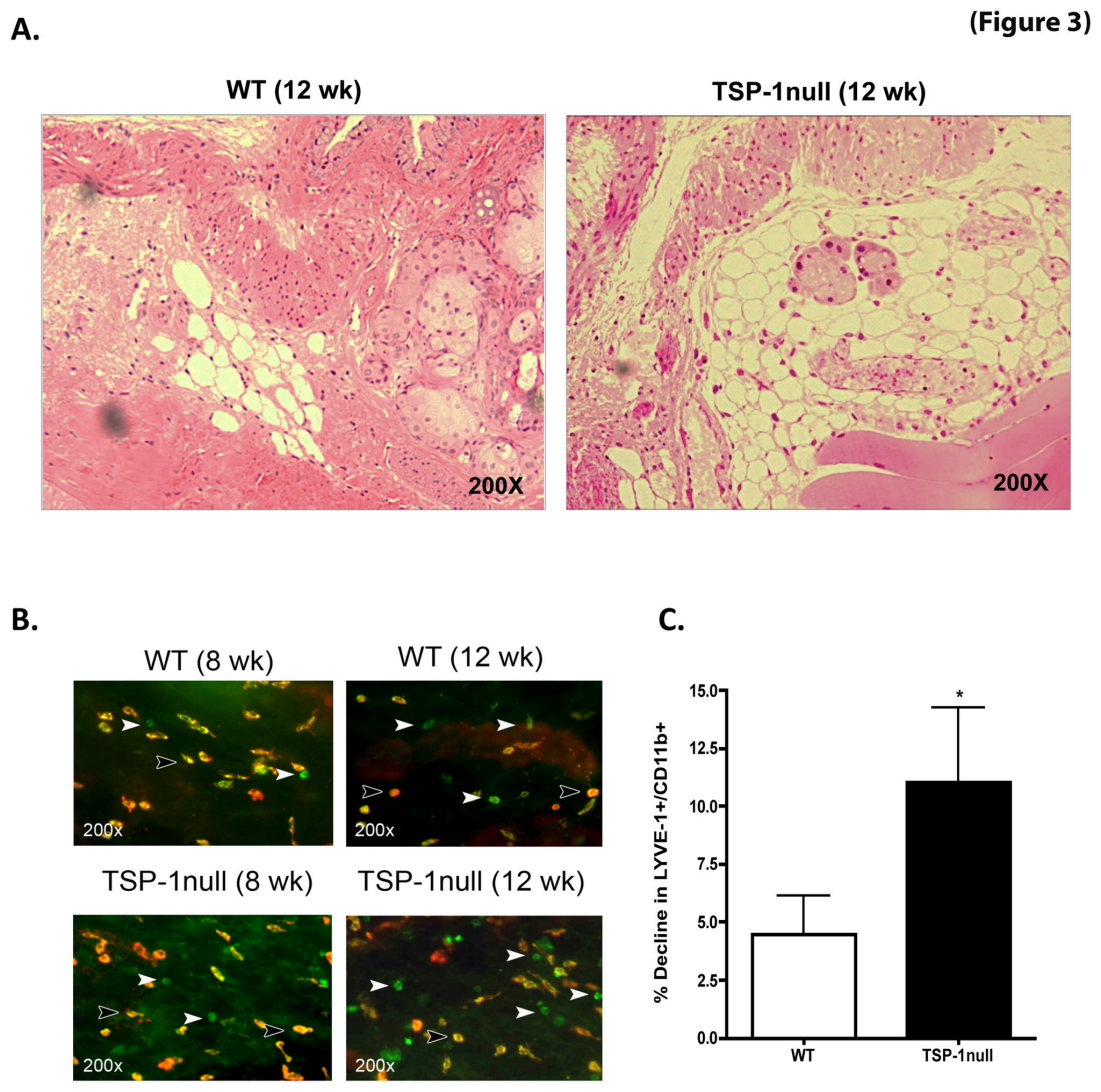
Increased lymphatic vessels in TSP-1 null conjunctiva are accompanied by a decline in LYVE-1^+^ macrophages. (**A**) Hematoxylin and eosin stained sections of the conjunctiva harvested from 12 week old WT or TSP-1 null mice show relatively increased lymphatic vessels in TSP-1 null conjunctiva. (**B**) Whole mounts of conjunctiva from 8 and 12 week old WT and TSP-1 null mice were immunostained with fluorochrome conjugated anti-LYVE-1(red) and anti-CD11b (green) antibodies. (**C**) Quantitative analysis of positively stained cells showed significantly increased decline in LYVE-1 expressing CD11b^+^ macrophages at 12 week (from 8 week) of age in TSP-1 null mice as compared to WT mice (* p < 0.05, compared to WT)..

As shown in Figure 3A, unlike blood vessels lymphatic vessels are discerned as vessels with thin endothelial walls and are devoid of blood cells. We detected relatively increased lymphatic vessels in H&E stained sections of TSP-1 null conjunctiva at 12 weeks (but not at 8 weeks), compared with the age-matched WT control tissue. To determine if this increase is reflected in a decline in LYVE-1 expressing monocytic cells we stained conjunctiva tissue and enumerated LIVE-1^+^ CD11b^+^ monocytic cells in WT and TSP-1 null conjunctiva, at both 8 and 12 weeks (Figure 3B). Quantitative analysis of immunofluorescence staining at these two ages demonstrated a greater decline in LYVE-1 expressing CD11b^+^ cells in TSP-1 null mice at 12 weeks, as compared to WT mice (Figure 3C). Thus our results support the histological observations in TSP-1 null conjunctiva indicating an increase in lymphatic vessels.

### The development of inflammatory effectors in the draining lymph nodes of TSP-1 null mice coincides with the increased lymphatic vessels

Resident APCs in any tissue, such as dendritic cells (DCs), are known to migrate via lymphatic vessels to the local draining lymph nodes where they present antigens to generate activated effector T cells. In an autoimmune disease such APCs are known to generate effectors that subsequently target the tissue. Both Th1 and Th17 represent inflammatory subsets of T effector cells that are associated with autoimmune responses in SS [25]. We next sought to determine the presence of such effectors in the CLN of WT or TSP-1 null mice as well as the target tissue.

**Figure 4:**
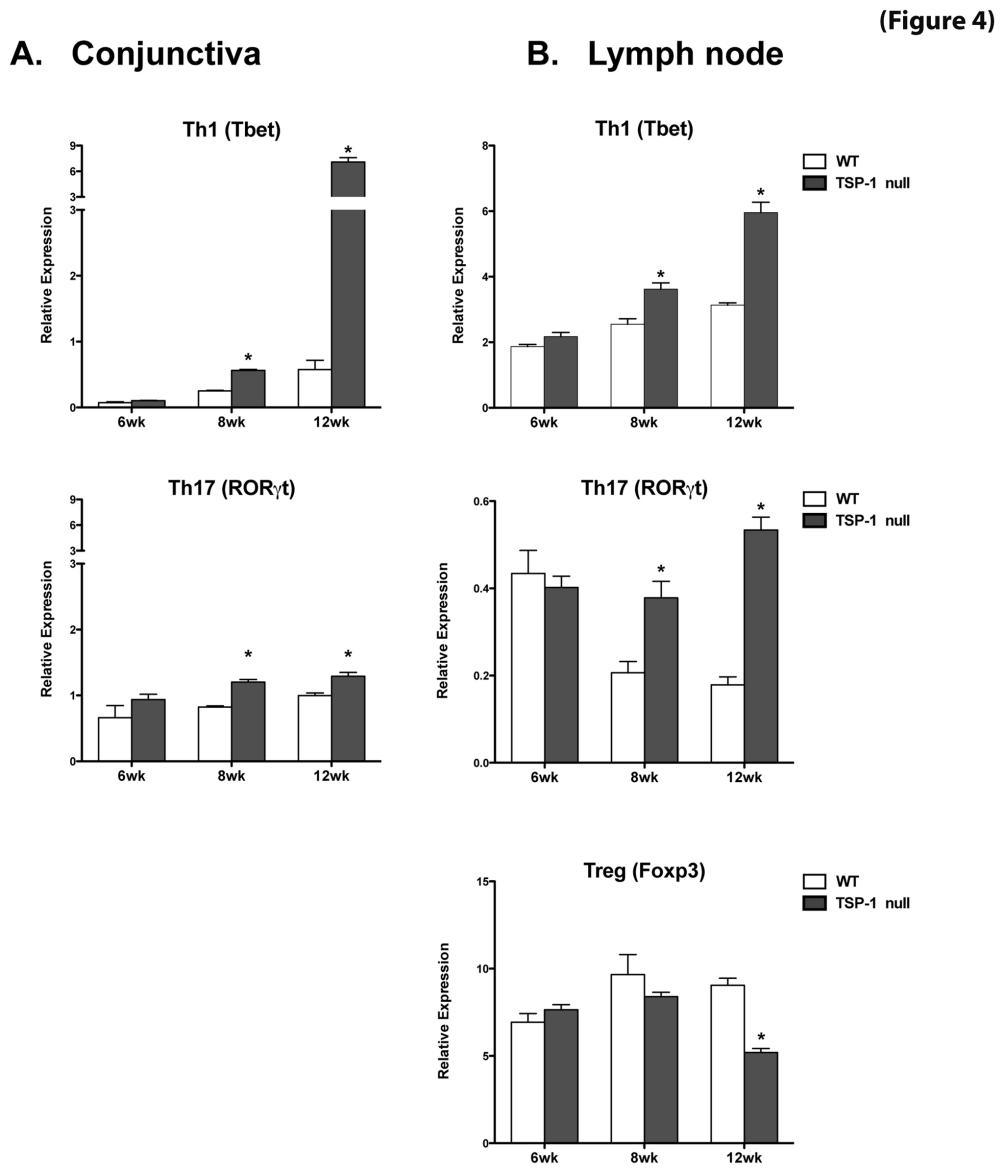
Inflammatory effectors are detected in the conjunctiva and the draining lymph nodes of TSP-1 null mice. Conjunctiva tissue and cervical lymph node cells were collected from WT and TSP-1 null mice at 6, 8, and 12 weeks (n=3 each). Extracted RNA was analyzed in a real-time PCR assay to determine the levels of message for the transcription factors Tbet, RORγt and Foxp3. No significant differences were detected in Tbet or RORγt expression in conjunctiva (**A**) and lymph nodes (**B**) at 6 weeks of age, while at 8 and 12 weeks the expression of both transcription factors was significantly increased in TSP-1 null mice compared with the WT control tissues. Expression of Foxp3 was significantly decreased in TSP-1 null lymph nodes at 12 weeks of age. Results are presented as relative expression of a transcription factor gene to that of the housekeeping gene GAPDH (* p <0.05 as compared to WT controls)..

Real-time PCR analysis using RNA isolated from WT or TSP-1 null CLN and conjunctiva was performed at 6, 8 and 12 weeks of age to assess expression levels of transcription factors Tbet (Th1) and RORγt (Th17). As shown in Figure 4, at 6 weeks of age no differences in Tbet or RORγt expression were detectable between WT and TSP-1 null mice in either conjunctiva or CLN. However, in 8 and 12 week old TSP-1 null mice the expression of both transcription factors was significantly increased in both conjunctiva and CLN as compared with the WT control tissues. Also expression levels of both Tbet and RORγt progressively increased with the age of mice.

Additionally we evaluated the expression of Foxp3 (Treg) in the CLN to determine if changes in this regulatory subset correspond to those in the inflammatory subsets. The expression of Foxp3 was significantly diminished in TSP-1 null CLN at 12 weeks of age. This observation is consistent with the previously reported reduced numbers of Foxp3+ Tregs in the spleen in TSP-1 null mice [9]. Together our results suggest that in TSP-1 null mice a peripheral imbalance in inflammatory and Treg cells results in the development of increased inflammatory effectors between 8 and 12 weeks that subsequently target the conjunctiva.

### Migration of dendritic cells carrying ocular surface antigens to the draining lymph nodes is enhanced in TSP-1 null mice

Antigen-presenting DCs are positioned as sentinels in the ocular surface, capturing foreign antigens. Under inflammatory conditions, DCs undergo a process called maturation, and readily relocate to CLN to stimulate the differentiation of naïve T cells into effectors cells [26]. Previously TSP-1 was reported to prevent maturation of DC [16], including corneal DCs [27]. Additionally, the expression of CCR7, a key molecule in DC trafficking to draining lymph nodes that is upregulated in mature DCs [28], was also reported as increased in TSP-1 deficient DCs [27]. These observations suggest an increased potential of ocular surface DCs in TSP-1 null mice to migrate to the draining lymph node. Such a possibility may then explain the increased levels of inflammatory effectors detected in TSP-1 null lymph nodes compared to their age-matched WT controls.

**Figure 5:**
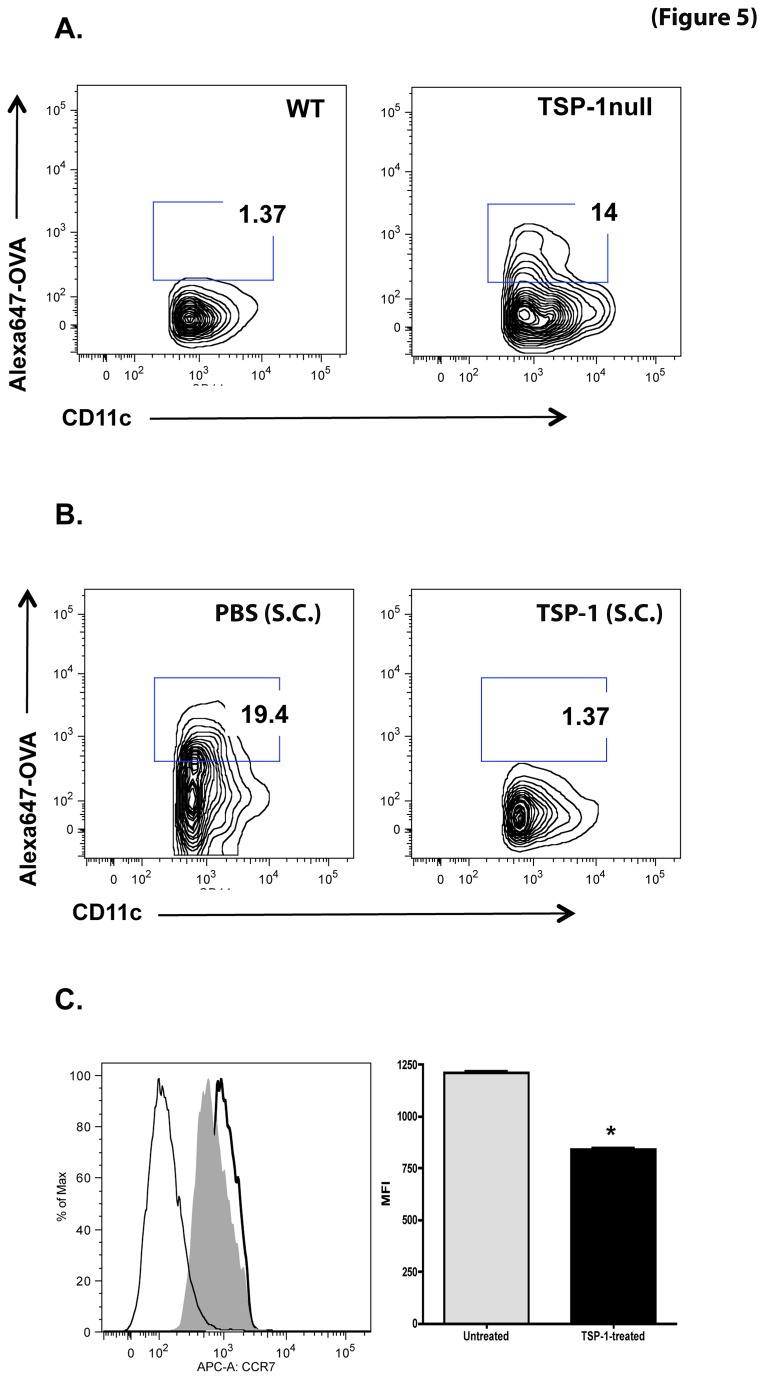
Migration of DCs carrying ocular surface antigens to draining LNs is enhanced in TSP-1 null mice. Cervical lymph nodes from 10-week old WT and TSP-1 null mice (n=3 each) were harvested 3 hr after topical application of Alexa647-conjugated OVA (125 µg/2.5 µl per eye). Lymph node cells were stained with fluorochrome-conjugated CD11c prior to analysis by flow cytometry and CD11c/Alexa647^+^ cells were identified. (**A**) A marked increase in CD11c/Alexa647^+^ cells was detectable in TSP-1 null lymph node cells compared to WT controls. (**B**) Blockade of CD11c/Alexa647^+^ dendritic cell migration to draining lymph nodes was observed in TSP-1 null mice following subconjunctival (S.C) injection of TSP-1 as compared to PBS-injected control group. (**C**) BMDC were treated with TSP-1 for 24 hr, stained with fluorochrome-conjugated CCR7, and analyzed by flow cytometry. The histogram shows fluorescence detected in unstained (thin line), untreated (thick line) and TSP-treated (filled grey) cells. The bar graph shows mean fluorescence intensity (MFI) of CCR7 staining. TSP-1 treatment significantly decreased the expression of CCR7 (* p<0.05).

To address this possibility we assessed the migration of resident TSP-1 null DCs carrying an antigen from the ocular surface to the CLN. We instilled Alexa-647-conjugated ovalbumin (OVA) as an antigen in the eyes of TSP-1 null and WT mice. Antigen-laden DCs (Alexa647^+^ CD11c^+^) migrating to the CLN were identified by flow cytometry. A significant increase in DCs was detected in TSP-1 null mice compared to WT controls (Figure 5A). These data clearly indicated an enhanced DC migration to the LN in TSP-1 null mice.

To determine if TSP-1 directly regulated CCR7 expression, we used bone marrow derived dendritic cells from WT mice and treated them *in vitro* with TSP-1 for 24 h and assessed their CCR7 expression by flow cytometry. We found that TSP-1 treatment significantly decreased the expression of CCR7 (Figure 5C). Based on these results we next determined if TSP-1 could block DC migration from TSP-1 null conjunctiva. We injected TSP-1 or PBS subconjunctivally before the application of Alexa-647-conjugated OVA to the ocular surface of TSP-1 null mice. Appearance of OVA-laden DCs was evaluated in the CLN. As shown in Figure 5B, a blockade of Alexa647^+^ CD11c^+^ DC migration to CLN was observed in TSP-1 null mice following TSP-1 injection as compared to PBS injection in the control group. Taken together, these data suggest that TSP-1 regulates DC migration from the ocular surface to the LN by regulating their expression of CCR7.

## Discussion

Ocular surface inflammation in TSP-1 deficient mice is associated with autoimmune SS and remarkably resembles histopathological and serological changes detectable in SS patients with KCS [9]. Despite appearing normal at birth, TSP-1 deficient mice progressively develop a chronic form of ocular surface disease with age. In this study we report that, as in SS, inflammatory damage in TSP-1 null mice is detected in the conjunctiva (in addition to the lacrimal gland and cornea as reported previously). This observation is supported by the tissue expression of inflammatory cytokines, the appearance of inflammatory infiltrates, the presence of increased lymphatic vessels and inflammatory Th1 and Th17 effectors as well as the loss of secretory function of mucin secreting goblet cells in TSP-1 null conjunctiva.

In patients with SS, abnormalities of lacrimal gland secretion compromise the protective function of tears, which in turn can affect tissues of the ocular surface, cornea and conjunctiva. Alterations in the morphology of epithelial cells in both of these tissues are noted in SS subjects [6,7,29]. Damage to epithelial cells of the cornea is reflected in a loss of their barrier function detectable by increased fluorescein staining [30]. Similarly, changes in ultrastructural morphology of apical conjunctival epithelial cells are reported in Sjögren’s patients [8]. The damage to conjunctival epithelium in SS also highlights an importance of the glycocalyx (mucin) layer in tears that protects the ocular surface from abrasions during lid movements. Our observations in TSP-1 null mice are consistent with these findings in that we note a metaplasia of conjunctival epithelial cells which is accompanied with reduced tear mucin levels as reported in SS patients [31].

The improved ocular signs in SS patients in response to anti-inflammatory treatment have supported a role of inflammation in the ocular surface damage seen in these patients [30]. In addition to histopathological changes the presence of inflammatory infiltrates was reported in the conjunctival biopsies of SS patients [6,21]. These infiltrates included neutrophils as well as lymphocytes with a predominance of T cells. Similar findings were also reported by evaluating conjunctival impression cytology specimens from SS patients [32]. Impression cytology represents a non- or minimally invasive biopsy of the ocular surface epithelium [33] that allows assessment of goblet cell density in addition to the cytologic features of the conjunctiva. Overexpression of several inflammatory markers were reported in conjunctival cells from impression cytology specimens of SS patients and these changes were also correlated with a significant decrease in goblet cells [34,35]. Thus it is believed that cytological changes in the conjunctiva of SS patients are likely due to increased expression of inflammatory mediators such as IL-6, IL-8, TNF-α and TGF-β1 [22,23]. However, the inflammatory effectors infiltrating the conjunctiva of SS patients are not yet as well characterized as those from the lacrimal glands. In TSP-1 null conjunctiva we detected increased expression of inflammatory cytokines associated with Th1 (IFN-γ and TNF-α) as well as Th17 (IL-6 and IL-17A) effectors and expression of these cytokines progressively increased with age. The presence of inflammatory mediators in TSP-1 null conjunctiva not only correlated with the abnormalities of the conjunctival epithelial cells but also with reduced goblet cell density as reported previously [9]. However, the source of the detected cytokines is not limited to adaptive immune cells as innate immune cells such as natural killer (NK), NKT or gamma delta T cells capable of producing these cytokines are also present in normal mouse conjunctiva [36,37]. It is quite likely that altered tear quality in TSP-1 null mice allows commensal microflora on the ocular surface to activate innate cells to produce proinflammatory cytokines. We also assessed the expression of transcription factors that are typically associated with Th1 (Tbet) and Th17 (RORγt) effectors (both in the conjunctiva and CLNs) and noted their significantly increased expression in the TSP-1 null conjunctiva at all ages while in CLNs after 8 weeks of age. However more recent reports indicate that in mucosal tissues NK cells or their subset are capable of expressing these transcription factors [38-40] although the presence of such a population in the conjunctiva is yet to be established. Since RORγt^+^ innate cells are rare in the peripheral lymph nodes our results suggest a more likely increase in Th1 and Th17 effectors in the CLNs of TSP-1 null mice, however a possible recruitment of NK cells to the draining lymph nodes as a part of an inflammatory response in these mice cannot be ruled out.

The presence of inflammatory effectors in the CLNs of TSP-1 null mice is consistent with the autoimmune pathology reported in these mice. Absence of these effectors at the age of 6 weeks and their detection from 8 weeks onwards suggests a possibility of sensitization to autoantigens during the interval of 6-8 weeks. Autoantigens derived from the ocular surface are likely presented during this period by mature DCs derived from inflamed conjunctiva. Increased inflammatory effectors in TSP-1 null conjunctiva and CLNs correlated with increased lymphatic vessels detectable in the conjunctiva, further suggesting the possibility of an enhanced egress of antigen-bearing DCs to the draining lymph nodes in TSP-1 null mice. This possibility is further supported by a previous report of significantly increased expression of CCR7, a draining lymph node homing receptor, by TSP-1 null DCs [27] and our results of *in vitro* inhibition of CCR7 in WT DCs by exogenous TSP-1. In this study we further provide *in vivo* evidence for such TSP-dependent regulation of DC migration from the ocular surface to the draining LNs. The antilymphangiogenic role of TSP-1 is also likely to contribute to such regulation [13]. Therefore, collectively, our results indicate that the exposure of DCs to TSP-1 in the tissue can prevent peripheral sensitization against tissue-derived autoantigens.

Interestingly a decline in Foxp3^+^ Tregs in the cervical CLNs of TSP-1 null mice was detectable at 12 weeks of age while such a decline in the spleen was previously reported at 8 weeks of age [9]. The reasons behind this difference and the earliest detection of Treg decline warrants further investigation. However, a decline in peripheral Treg population concomitant with an increase in inflammatory effectors is consistent with the autoimmune pathogenesis of the inflammatory changes in TSP-1 null mice. These observations can also be explained by the compromised ability of TSP-1 null APCs to induce Foxp3^+^ Tregs as detected in our *in vitro* experiments (Mir, F. et al., manuscript submitted).

Thus our results shed light on possible immunologic mechanisms that contribute to the development of autoimmune SS. Similar to the lacrimal gland in TSP-1 null mice, the loss of secretory function of goblet cells in the conjunctiva appeared prior to its infiltration by inflammatory cells detectable by histology. However, innate cells resident in the conjunctiva are capable of responding to microbial stimuli from the ocular surface resulting in an inflammatory cytokine milieu that interferes with goblet cell function and promotes DC migration. In addition to shared epitopes between ocular tissues and the lacrimal gland [41] the common draining lymph nodes between these tissues likely generate a closely related immune response. Our observations in TSP-1 null mice indicate that the inflammatory processes at the ocular surface and lacrimal gland are temporally distinct, presumably due to greater vulnerability of the former tissue to pathogens. The decreased tear MUC5AC levels further alter the barrier against pathogens amplifying the local inflammatory process. Autoantigens derived from the damaged ocular surface are readily presented by local DCs to activate inflammatory effectors in the draining LNs, which target ocular surface tissue to contribute further to an ongoing inflammation and related tissue damage.

In conclusion, this study demonstrates that TSP-1 deficient mice develop inflammation in the conjunctiva and have reduced goblet cell secretion similar to that seen in patients with SS. This model provides a better understanding of the disease, allowing the investigation of SS development in ways that would be inaccessible in a human patient. Furthermore our results suggest that the anti-inflammatory and anti-lymphangiogenic properties of TSP-1 contribute to the regulation of an autoimmune response. Thus TSP-1 holds a strong therapeutic potential in the treatment of ocular surface inflammation associated with autoimmune SS.

## References

[1] Kassan SS, Moutsopoulos HM (2004) Clinical manifestations and early diagnosis of Sjogren syndrome. Arch Intern Med 164: 1275-1284. doi:10.1001/archinte.164.12.1275. PubMed: 15226160. 15226160 10.1001/archinte.164.12.1275

[2] Jonsson R, Vogelsang P, Volchenkov R, Espinosa A, Wahren-Herlenius M et al. (2011) The complexity of Sjogren’s syndrome: novel aspects on pathogenesis. Immunol Lett 141: 1-9. doi:10.1016/j.imlet.2011.06.007. PubMed: 21777618. 21777618 10.1016/j.imlet.2011.06.007

[3] Hu Y, Nakagawa Y, Purushotham KR, Humphreys-Beher MG (1992) Functional changes in salivary glands of autoimmune disease-prone NOD mice. Am J Physiol 263: E607-E614. PubMed: 1415679. 1415679 10.1152/ajpendo.1992.263.4.E607

[4] Hoffman RW, Alspaugh MA, Waggie KS, Durham JB, Walker SE (1984) Sjogren’s syndrome in MRL/l and MRL/n mice. Arthritis Rheum 27: 157-165. doi:10.1002/art.1780270206. PubMed: 6421291. 6421291 10.1002/art.1780270206

[5] Nguyen CQ, Kim H, Cornelius JG, Peck AB (2007) Development of Sjogren’s syndrome in nonobese diabetic-derived autoimmune-prone C57BL/6.NOD-Aec1Aec2 mice is dependent on complement component-3. J Immunol 179: 2318-2329. PubMed: 17675493. 17675493 10.4049/jimmunol.179.4.2318 PMC2850056

[6] Raphael M, Bellefqih S, Piette JC, Le Hoang P, Debre P et al. (1988) Conjunctival biopsy in Sjogren’s syndrome: correlations between histological and immunohistochemical features. Histopathology 13: 191-202. doi:10.1111/j.1365-2559.1988.tb02024.x. PubMed: 3169687. 3169687 10.1111/j.1365-2559.1988.tb02024.x

[7] Wakamatsu TH, Sato EA, Matsumoto Y, Ibrahim OM, Dogru M et al. (2010) Conjunctival in vivo confocal scanning laser microscopy in patients with Sjogren syndrome. Invest Ophthalmol Vis Sci 51: 144-150. doi:10.1167/iovs.08-2722. PubMed: 19696170. 19696170 10.1167/iovs.08-2722

[8] Koufakis DI, Karabatsas CH, Sakkas LI, Alvanou A, Manthos AK et al. (2006) Conjunctival surface changes in patients with Sjogren’s syndrome: a transmission electron microscopy study. Invest Ophthalmol Vis Sci 47: 541-544. doi:10.1167/iovs.05-0804. PubMed: 16431948. 16431948 10.1167/iovs.05-0804

[9] Turpie B, Yoshimura T, Gulati A, Rios JD, Dartt DA et al. (2009) Sjogren’s syndrome-like ocular surface disease in thrombospondin-1 deficient mice. Am J Pathol 175: 1136-1147. doi:10.2353/ajpath.2009.081058. PubMed: 19700744. 19700744 10.2353/ajpath.2009.081058 PMC2731132

[10] Adams JC, Lawler J (2011) The thrombospondins. Cold Spring Harb Perspect Biol 3: a009712. doi:10.1101/cshperspect.a009712. PubMed: 21875984. 21875984 10.1101/cshperspect.a009712 PMC3179333

[11] Sekiyama E, Nakamura T, Cooper LJ, Kawasaki S, Hamuro J et al. (2006) Unique distribution of thrombospondin-1 in human ocular surface epithelium. Invest Ophthalmol Vis Sci 47: 1352-1358. doi:10.1167/iovs.05-1305. PubMed: 16565368. 16565368 10.1167/iovs.05-1305

[12] Lawler J (2000) The functions of thrombospondin-1 and-2. Curr Opin Cell Biol 12: 634-640. doi:10.1016/S0955-0674(00)00143-5. PubMed: 10978901. 10978901 10.1016/s0955-0674(00)00143-5

[13] Cursiefen C, Maruyama K, Bock F, Saban D, Sadrai Z et al. (2011) Thrombospondin 1 inhibits inflammatory lymphangiogenesis by CD36 ligation on monocytes. J Exp Med 208: 1083-1092. doi:10.1084/jem.20092277. PubMed: 21536744. 21536744 10.1084/jem.20092277 PMC3092349

[14] Sarfati M, Fortin G, Raymond M, Susin S (2008) CD47 in the immune response: role of thrombospondin and SIRP-alpha reverse signaling. Curr Drug Targets 9: 842-850. doi:10.2174/138945008785909310. PubMed: 18855618. 18855618 10.2174/138945008785909310

[15] Grimbert P, Bouguermouh S, Baba N, Nakajima T, Allakhverdi Z et al. (2006) Thrombospondin/CD47 interaction: a pathway to generate regulatory T cells from human CD4+ CD25- T cells in response to inflammation. J Immunol 177: 3534-3541. PubMed: 16951312. 16951312 10.4049/jimmunol.177.6.3534

[16] Doyen V, Rubio M, Braun D, Nakajima T, Abe J et al. (2003) Thrombospondin 1 is an autocrine negative regulator of human dendritic cell activation. J Exp Med 198: 1277-1283. doi:10.1084/jem.20030705. PubMed: 14568985. 14568985 10.1084/jem.20030705 PMC2194231

[17] Avice MN, Rubio M, Sergerie M, Delespesse G, Sarfati M (2000) CD47 ligation selectively inhibits the development of human naive T cells into Th1 effectors. J Immunol 165: 4624-4631. PubMed: 11035105. 11035105 10.4049/jimmunol.165.8.4624

[18] Zamiri P, Masli S, Kitaichi N, Taylor AW, Streilein JW (2005) Thrombospondin plays a vital role in the immune privilege of the eye. Invest Ophthalmol Vis Sci 46: 908-919. doi:10.1167/iovs.04-0362. PubMed: 15728547. 15728547 10.1167/iovs.04-0362

[19] Masli S, Turpie B, Streilein JW (2006) Thrombospondin orchestrates the tolerance-promoting properties of TGFbeta-treated antigen-presenting cells. Int Immunol 18: 689-699. PubMed: 16569680. 16569680 10.1093/intimm/dxl006

[20] Nguyen CQ, Peck AB (2009) Unraveling the pathophysiology of Sjogren syndrome-associated dry eye disease. Ocul Surf 7: 11-27. doi:10.1016/S1542-0124(12)70289-6. PubMed: 19214349. 19214349 10.1016/s1542-0124(12)70289-6 PMC2861866

[21] Stern ME, Gao J, Schwalb TA, Ngo M, Tieu DD et al. (2002) Conjunctival T-cell subpopulations in Sjogren’s and non-Sjogren’s patients with dry eye. Invest Ophthalmol Vis Sci 43: 2609-2614. PubMed: 12147592. 12147592

[22] Pflugfelder SC, Jones D, Ji Z, Afonso A, Monroy D (1999) Altered cytokine balance in the tear fluid and conjunctiva of patients with Sjogren’s syndrome keratoconjunctivitis sicca. Curr Eye Res 19: 201-211. doi:10.1076/ceyr.19.3.201.5309. PubMed: 10487957. 10487957 10.1076/ceyr.19.3.201.5309

[23] Yoon KC, Jeong IY, Park YG, Yang SY (2007) Interleukin-6 and tumor necrosis factor-alpha levels in tears of patients with dry eye syndrome. Cornea 26: 431-437. doi:10.1097/ICO.0b013e31803dcda2. PubMed: 17457192. 17457192 10.1097/ICO.0b013e31803dcda2

[24] Chen L, Cursiefen C, Barabino S, Zhang Q, Dana MR (2005) Novel expression and characterization of lymphatic vessel endothelial hyaluronate receptor 1 (LYVE-1) by conjunctival cells. Invest Ophthalmol Vis Sci 46: 4536-4540. doi:10.1167/iovs.05-0975. PubMed: 16303945. 16303945 10.1167/iovs.05-0975 PMC1397798

[25] Katsifis GE, Moutsopoulos NM, Wahl SM (2007) T lymphocytes in Sjogren’s syndrome: contributors to and regulators of pathophysiology. Clin Rev Allergy Immunol 32: 252-264. doi:10.1007/s12016-007-8011-8. PubMed: 17992592. 17992592 10.1007/s12016-007-8011-8

[26] Schlereth S, Lee HS, Khandelwal P, Saban DR (2012) Blocking CCR7 at the ocular surface impairs the pathogenic contribution of dendritic cells in allergic conjunctivitis. Am J Pathol 180: 2351-2360. doi:10.1016/j.ajpath.2012.02.015. PubMed: 22507838. 22507838 10.1016/j.ajpath.2012.02.015 PMC5691338

[27] Saban DR, Bock F, Chauhan SK, Masli S, Dana R (2010) Thrombospondin-1 derived from APCs regulates their capacity for allosensitization. J Immunol 185: 4691-4697. doi:10.4049/jimmunol.1001133. PubMed: 20844200. 20844200 10.4049/jimmunol.1001133 PMC3090006

[28] Saeki H, Moore AM, Brown MJ, Hwang ST (1999) Cutting edge: secondary lymphoid-tissue chemokine (SLC) and CC chemokine receptor 7 (CCR7) participate in the emigration pathway of mature dendritic cells from the skin to regional lymph nodes. J Immunol 162: 2472-2475. PubMed: 10072485. 10072485

[29] Villani E, Galimberti D, Viola F, Mapelli C, Ratiglia R (2007) The cornea in Sjogren’s syndrome: an in vivo confocal study. Invest Ophthalmol Vis Sci 48: 2017-2022. doi:10.1167/iovs.06-1129. PubMed: 17460255. 17460255 10.1167/iovs.06-1129

[30] Hyon JY, Lee YJ, Yun PY (2007) Management of ocular surface inflammation in Sjogren syndrome. Cornea 26: S13-S15. doi:10.1097/ICO.0b013e31812f6782. PubMed: 17881909. 17881909 10.1097/ICO.0b013e31812f6782

[31] Argüeso P, Balaram M, Spurr-Michaud S, Keutmann HT, Dana MR et al. (2002) Decreased levels of the goblet cell mucin MUC5AC in tears of patients with Sjogren syndrome. Invest Ophthalmol Vis Sci 43: 1004-1011. PubMed: 11923240. 11923240

[32] Pflugfelder SC, Huang AJ, Feuer W, Chuchovski PT, Pereira IC et al. (1990) Conjunctival cytologic features of primary Sjogren’s syndrome. Ophthalmology 97: 985-991. PubMed: 1698273. 1698273 10.1016/s0161-6420(90)32478-8

[33] Calonge M, Diebold Y, Sáez V, Enríquez de Salamanca A, García-Vázquez C et al. (2004) Impression cytology of the ocular surface: a review. Exp Eye Res 78: 457-472. doi:10.1016/j.exer.2003.09.009. PubMed: 15106925. 15106925 10.1016/j.exer.2003.09.009

[34] Pisella PJ, Brignole F, Debbasch C, Lozato PA, Creuzot-Garcher C et al. (2000) Flow cytometric analysis of conjunctival epithelium in ocular Rosaceae and keratoconjunctivitis sicca. Ophthalmology 107: 1841-1849. doi:10.1016/S0161-6420(00)00347-X. PubMed: 11013183. 11013183 10.1016/s0161-6420(00)00347-x

[35] Brignole F, Pisella PJ, Goldschild M, De Saint Jean M, Goguel A et al. (2000) Flow cytometric analysis of inflammatory markers in conjunctival epithelial cells of patients with dry eyes. Invest Ophthalmol Vis Sci 41: 1356-1363. PubMed: 10798650. 10798650

[36] Rachitskaya AV, Hansen AM, Horai R, Li Z, Villasmil R et al. (2008) Cutting edge: NKT cells constitutively express IL-23 receptor and RORgammat and rapidly produce IL-17 upon receptor ligation in an IL-6-independent fashion. J Immunol 180: 5167-5171. PubMed: 18390697. 18390697 10.4049/jimmunol.180.8.5167 PMC2442579

[37] Zhang X, Volpe EA, Gandhi NB, Schaumburg CS, Siemasko KF et al. (2012) NK cells promote Th-17 mediated corneal barrier disruption in dry eye. PLOS ONE 7: e36822. doi:10.1371/journal.pone.0036822. PubMed: 22590618. 22590618 10.1371/journal.pone.0036822 PMC3348128

[38] Reynders A, Yessaad N, Vu Manh TP, Dalod M, Fenis A et al. (2011) Identity, regulation and in vivo function of gut NKp46+RORgammat+ and NKp46+RORgammat- lymphoid cells. EMBO J 30: 2934-2947. doi:10.1038/emboj.2011.201. PubMed: 21685873. 21685873 10.1038/emboj.2011.201 PMC3160256

[39] Vonarbourg C, Mortha A, Bui VL, Hernandez PP, Kiss EA et al. (2010) Regulated expression of nuclear receptor RORgammat confers distinct functional fates to NK cell receptor-expressing RORgammat(+) innate lymphocytes. Immunity 33: 736-751. doi:10.1016/j.immuni.2010.10.017. PubMed: 21093318. 21093318 10.1016/j.immuni.2010.10.017 PMC3042726

[40] Powell N, Canavan JB, MacDonald TT, Lord GM (2010) Transcriptional regulation of the mucosal immune system mediated by T-bet. Mucosal Immunol 3: 567-577. doi:10.1038/mi.2010.53. PubMed: 20844482. 20844482 10.1038/mi.2010.53

[41] Yu D, Thelin WR, Randell SH, Boucher RC (2012) Expression profiles of aquaporins in rat conjunctiva, cornea, lacrimal gland and Meibomian gland. Exp Eye Res 103: 22-32. doi:10.1016/j.exer.2012.07.005. PubMed: 22828047. 22828047 10.1016/j.exer.2012.07.005

